# Breast cancer-specific survival among immigrants and non-immigrants invited to BreastScreen Norway

**DOI:** 10.1016/j.jmh.2024.100222

**Published:** 2024-03-05

**Authors:** Sameer Bhargava, Jonas Gjesvik, Jonas Thy, Marthe Larsen, Solveig Hofvind

**Affiliations:** a Cancer Registry of Norway, Norwegian Institute of Public Health, Oslo, Norway; b Department of Oncology, Akershus University Hospital, Lørenskog, Norway; c Department of Health and Care Sciences, Faculty of Health Sciences, The Arctic University of Norway, Tromsø, Norway

**Keywords:** Migration, Minority health, Breast cancer, Mammographic screening

## Abstract

**Introduction:**

We have previously shown that immigrants have lower attendance in BreastScreen Norway than non-immigrants and that non-Western immigrants have lower incidence of breast cancer, but more advanced disease.

**Purpose:**

To compare breast cancer-specific survival for immigrants versus non-immigrants diagnosed with screen-detected or symptomatic breast cancer.

**Material and methods:**

We analyzed data from 28,320 women aged 50–69 diagnosed with breast cancer after being invited to BreastScreen Norway. We divided women into three groups; non-immigrants, immigrants from Western countries and immigrants from non-Western countries. We stratified our analyses according to detection mode (screen-detected breast cancer, interval cancer and cancer detected outside screening), and used cox regression to model the association between immigrants/non-immigrants and time to breast cancer death.

**Results:**

Among screen-detected breast cancers, 28.7% were histologic grade 3 among immigrants from non-Western countries compared to 21.3% among non-immigrants. Interval cancers and cancers detected outside screening had larger tumor diameter and a higher percentage were histologic grade 3 and lymph node positive among immigrants from non-Western countries compared to non-immigrants. Hazard ratio (95% confidence interval) adjusted for age and year of diagnosis for time to breast cancer death compared to non-immigrants was 0.70 (0.39–1.27) for immigrants from Western countries and 0.52 (0.23–1.17) for immigrants from non-Western countries.

**Conclusion:**

Despite more advanced histopathological tumor characteristics among immigrants from non-Western countries compared to non-immigrants, we did not observe statistically significant differences in breast-cancer specific survival between the two groups. Keeping in mind the low number of breast cancer deaths and possible overestimation of survival among immigrants, this might imply that equity in outcome can be achieved through adequate follow-up and treatment despite inequal access.

## 1 Introduction

Breast cancer is the most frequent cancer diagnosed among women in most countries (Bray et al., 2018). However, the incidence and mortality from the disease vary geographically. While the incidence of breast cancer is higher in Western versus non-Western countries, differences in mortality are less pronounced, and the highest mortality is reported in non-Western regions (Bray et al., 2018).

There are limited studies on breast cancer death or survival among immigrants. The few studies performed have shown varying results and have often been considered non-conclusive. Studies showing changes in mortality from lower to higher among immigrants compared to non-immigrants (Abdoli et al., 2015) could reflect sociodemographic variation over time within the immigrant population, including changes in countries where immigrants migrate from and in reasons for migration. There might also be differences between age groups, with younger immigrants having poorer breast cancer survival than non-immigrants, and older immigrants having better survival (Beiki et al., 2012). Further, differences in survival between immigrants and non-immigrants may also be observed when stratifying women by menopausal status and tumor histology (Mousavi et al., 2013).

Studies with data from Norway have shown higher incidence of breast cancer among immigrants from the Nordic countries and Western Europe compared to non-immigrants, and lower incidence of breast cancer among immigrants from non-Western countries (Hjerkind et al., 2017). Further, these studies have revealed more advanced disease, lower breast cancer survival and younger age at diagnosis among groups of immigrant women from non-Western countries diagnosed with breast cancer in Norway (Latif et al., 2015; Thogersen et al., 2017; Thøgersen et al., 2018). However, for most non-Western immigrant groups, differences in breast cancer survival for immigrants versus non-immigrants did not differ statistically significantly (Thøgersen et al., 2018).

Breast cancer screening aims to reduce breast cancer mortality through early detection of the disease (European Commission Initiative on Breast Cancer 2022). Women with screen-detected breast cancer have favorable tumor characteristics and better survival from breast cancer than those with interval cancer or breast cancer diagnosed outside organized screening (Hofvind et al., 2016).

BreastScreen Norway invites all women aged 50–69 for mammographic screening every second year (Hofvind et al., 2017). Screening is performed at 17 stationary units across the country as well as four mobile units. The women receive an invitation letter with a predefined but changeable appointment time and an information sheet written in Norwegian, regardless of their country of birth. BreastScreen Norway is currently conducting a randomized controlled trial, examining whether translating the invitation letter and information sheet to the main language of selected countries of birth will impact attendance (Cancer Registry of Norway 2022).

We have previously shown that immigrants in Norway have lower attendance rates than non-immigrants (Bhargava et al., 2017). Immigrants have not only been shown to have lower uptake of breast cancer screening, but also for cervical cancer and colorectal cancer screening compared to non-immigrants (Leinonen et al., 2017; Botteri et al., 2022). Among women who attend BreastScreen Norway, we have observed less favorable tumor characteristics among immigrants versus non-immigrants (Thy et al., 2022). However, as far as we are aware no studies have presented results on breast cancer-specific survival by detection mode for immigrants versus non-immigrants among women in the age group targeted for breast cancer screening. Such knowledge is important in order to get a better understanding of the performance of breast cancer screening for immigrants and non-immigrants.

The United Nation's Sustainable Development Goal (SDG) 3 aims to “ensure healthy lives and promote well-being for all at all ages” (United Nations Development of Economic and Social Affairs 2023). Targets of SDG 3 include to reduce mortality from cancer and other non-communicable disease through prevention and treatment, and to ensure universal health coverage. Further, the Nordic welfare model emphasizes equity in access for the entire population (Magnussen et al., 2009). This study would thus fill an important knowledge gap as differences in breast-cancer specific survival could be in conflict both with SDG 3 and the Nordic welfare model.

In this registry-based nationwide, population-based cohort study, we compared histopathological tumor characteristics and breast cancer-specific survival for women with screen-detected breast cancer, interval cancer and breast cancer detected outside BreastScreen Norway among immigrants versus non-immigrants. We expected lower breast cancer-specific survival among immigrants from non-Western countries compared to non-immigrants and had no reason to expect that difference in survival varied according to detection mode.

## 2 Material and methods

### 2.1 Data sources and variables

The study was approved by the data protection officer for research at Oslo University Hospital (20/12601).

We received data on birth, death, cause of death, emigration, country of birth, screening examinations (invitations, attendance and outcomes) and tumors (date of diagnosis and histopathological tumor characteristics) from the Cancer Registry of Norway. The Cancer Registry of Norway receives information on death and cause of death from the national Cause of Death Registry (Larsen et al., 2019). Information about country of birth is registered at the National Population Register, and has been available in the Cancer Registry of Norway's databases since 2018 (Vinberg et al., 2019).

We followed women aged 50–69 invited to BreastScreen Norway and diagnosed with invasive breast cancer during the period from January 1st 1996 to December 31st 2019. Women were followed from the date of diagnosis until death, emigration, or end of study period. We excluded women with no information on country of birth (*n* = 286) and women with no follow-up time (diagnosis from death certificate only, *n* = 16).

Women born outside Norway were defined as immigrants and classified according to their country of birth. Women born in Norway were classified as non-immigrants. Immigrants were divided into two groups; immigrants from Western countries and immigrants from non-Western countries (Appendix). Western countries included countries in Western Europe, Northern America, Australia and New Zealand, while non-Western countries included all other countries. This three-level categorical variable is referred to as “birth country groups”.

Detection mode was based on primary diagnosis of invasive breast cancer. Screen-detected breast cancer was defined as breast cancer diagnosed within six months after a positive screening examination. Interval cancer was defined as breast cancer diagnosed after a negative screening examination or 6 to 24 months after a false positive screening exam. Breast cancer detected outside the screening program was defined as breast cancer diagnosed among women invited but never attended or breast cancer diagnosed more than two years after the last screening examination.

Histopathological tumor characteristics included type (invasive carcinoma of no special type (NST), lobular or other invasive), tumor diameter (mm), histologic grade (1, 2 or 3), and lymph node status (positive/negative) (Goldhirsch et al., 2013).

Breast cancer is categorized as stage I–IV. Stages I–III are considered potentially curable, while women with stage IV breast cancer have metastatic disease that has spread to other organs, and cure is not considered possible. In order to differentiate between potentially curable and incurable disease, we categorized stage according to absence (stages I-III) or presence (stage IV) of metastases.

Age (years), follow-up time (years) and tumor diameter (mm) were continuous variables. All other variables were categorical. The outcome of interest (dependent variable) was breast cancer death. The remaining variables were considered independent variables that could suggest cause of breast cancer death.

### 2.2 Statistical analysis

Descriptive results were presented as frequencies and percentages for categorical variables, and means with standard deviations (SD), or medians with interquartile range (IQR) for continuous variables. Unadjusted and adjusted hazard ratios (HR) with 95% confidence intervals (CI) from cox regression were presented to show the association between birth country groups and time to breast cancer death. Adjusted models included age at diagnosis and year of diagnosis in addition to the birth country group variable. . All analyses were performed using Stata version 17.0 (StataCorp).

## 3 Results

The study included 28,320 women diagnosed with invasive breast cancer; 26,501 non-immigrants (93.6%), 841 immigrant women from Western countries (3.0%), and 978 immigrant women from non-Western countries (3.4%) during the period from January 1st 1996 to December 31st 2019 (Table 1). Mean age at diagnosis was 60.2 years (SD=5.6) for non-immigrants, 59.6 years (SD=5.6) for women born in Western countries and 58.3 years (SD=5.5) for women from non-Western countries.

**Table 1:** Age at diagnosis, follow-up time, mode of detection and outcomes stratified by birth country group, 1996–2019.

	All women		Non-immigrants		Western		Non-Western	
	*n* = 28,320		*n* = 26,501		*n* = 841		*n* = 978	
Age at diagnosis (mean, SD)	60.1	5.6	60.2	5.6	59.6	5.6	58.3	5.5
Follow-up time, years (median, IQR)	8.5	5.1–17.0	8.7	5.2–17.1	7.1	4.8–17.0	5.1	2.8–11.8
	n	%	n	%	n	%	n	%
Mode of detection								
Screen detected cancers	10,739	37.9	10,179	38.4	268	31.9	292	29.9
Interval cancers	3642	12.9	3437	13.0	113	13.4	92	9.4
Outside screening	13,939	49.2	12,885	48.6	460	54.7	594	60.7
Outcome								
Death from breast cancer	3519	12.4	3318	12.5	109	13.0	92	9.4
Death from other causes	1767	6.2	1675	6.3	54	6.4	38	3.9
Emigrated	171	0.6	70	0.3	79	9.4	22	2.3
Alive at end of follow-up	22,863	80.8	21,438	80.9	599	71.2	826	84.5

Abbreviations: SD - Standard deviation. IQR - Inter quartile range.

Among non-immigrants, 38.4% of the cancers were screen-detected, 13.0% were interval cancer and 48.6% were detected outside screening (Table 1). Among women from Western countries 54.7% of the cancers were detected outside screening, while 60.7% of cancers among women from non-Western countries were detected outside screening. A total of 3318 breast cancer deaths was observed for non-immigrants, 109 for women from Western countries and 92 for women from non-Western countries.

For screen-detected cancers, 28.7% of tumors among immigrants from non-Western countries were histologic grade 3 compared to 21.3% among non-immigrants (Table 2). For both interval cancers and cancers detected outside screening, tumors among non-Western immigrants had larger tumor diameters compared to non-immigrants. Also, for interval cancers, the percentage of histologic grade 3 tumors was 46.2% among immigrants from non-Western countries, compared to 38.3% among non-immigrants. For cancers outside screening, the respective percentage of histologic grade 3 tumors and lymph node positive tumors were 42.2% and 63.7% among immigrants from non-Western countries, compared to 33.0% and 58.3% among non-immigrants (Table 2).

**Table 2:** Histopathological tumor characteristics for women with screen-detected breast cancers, interval cancers and cancers detected outside screening among non-immigrants, immigrants from Western countries and immigrants from non-Western countries between 1996 and 2019.

	Screen detected cancers						Interval cancers						Cancers outside the program					
	Non-immigrants		Western		Non-Western		Non-immigrants		Western		Non-Western		Non-immigrants		Western		Non-Western	
	*n* = 10,179		*n* = 268		*n* = 292		*n* = 3437		*n* = 113		*n* = 92		*n* = 12,885		*n* = 460		*n* = 594	
	10,179.00		268		292.00		3437		113		92		12,885		460		594	
Tumor diameter, mm (median, IQR)	13 (9–19)		12 (8–20)		13 (9–21)		19 (13–25)		18 (12–25)		21 (13–27)		19 (13–25)		18 (13–25)		21 (14–30)	
Data not available (n)	235		11		12		336		10		8		7934		258		242	
	N	%	n	%	n	%	n	%	n	%	n	%	n	%	n	%	n	%
Morfologic type																		
Invasive ductal carcinoma NST	8721	85.7	221	82.5	246	84.3	2859	83.2	97	85.8	81	88.0	11,024	85.6	402	87.4	516	86.9
Invasive lobular carcinoma	973	9.6	35	13.1	33	11.3	421	12.3	13	11.5	8	8.7	1296	10.1	44	9.6	55	9.3
Other Invasive	485	4.8	12	4.5	13	4.5	157	4.6	3	2.7	3	3.3	565	4.4	14	3.0	23	3.9
Histologic Grade																		
1	3118	31.2	93	35.5	60	21.3	540	16.3	21	19.3	7	7.7	2285	19.8	79	19.5	67	13.1
2	4745	47.5	120	45.8	141	50.0	1508	45.4	54	49.5	42	46.2	5443	47.2	186	45.9	228	44.7
3	2125	21.3	49	18.7	81	28.7	1271	38.3	34	31.19	42	46.2	3809	33.0	140	34.6	215	42.2
Data not available (n)	191		6		10		118		4		1		1348		55		84	
Lymph node status																		
Positive	2500	25.2	63	24.4	74	26.1	1350	41.6	41	38.0	37	43.0	2583	58.3	100	55.0	209	63.7
Data not available (n)	249		10		8		190		5		6		8452		278		266	

Abbreviations: IQR - Interquartile range. NST - No special type.

**Fig. 1:**
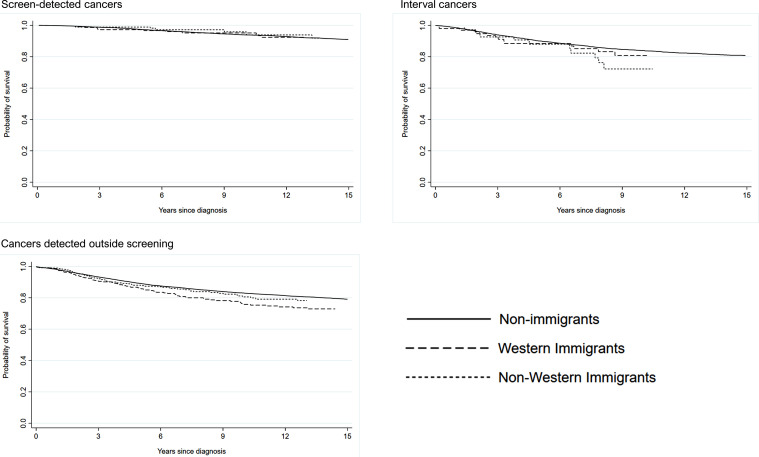
Breast cancer-specific survival among non-immigrants, immigrants from Western countries and immigrants from non-Western countries, stratified by cancer detection mode.

We observed no statistically significant differences in breast cancer-specific survival between birth country groups (Fig. 1, Table 3). Hazard ratio (95% confidence interval) adjusted for age and year of diagnosis for time to breast cancer death compared to non-immigrants was 0.70 (0.39–1.27) for immigrants from Western countries and 0.52 (0.23–1.17) for immigrants from non-Western countries.

**Table 3:** Unadjusted and adjusted hazard ratios and 95% confidence intervals for association between birth country groups and time to breast cancer death for all cancers.

	Hazard ratio		
	Estimate	95%CI	p
**Unadjusted**			
Non-Immigrants	ref		
Western	0.72	0.40–1.30	0.28
Non-Western	0.48	0.22–1.08	0.08
**Adjusted for age and year of diagnosis**			
Non-Immigrants	ref		
Western	0.70	0.39–1.27	0.24
Non-Western	0.52	0.23–1.17	0.12

We did not stratify cancers with metastatic disease by detection mode due to a low number of cases. Among all cases, 665 (2.6%) non-immigrants, 23 (2.8%) immigrants from Western countries and 26 (2.7%) immigrants from non-Western countries had metastatic disease at time of diagnosis.

## 4 Discussion

Breast cancer among immigrants from non-Western countries tended to be histopathologically less prognostically favorable compared to non-immigrants regardless of detection mode. Despite these findings, we did not observe statically significant differences breast cancer-specific survival among immigrants compared to non-immigrants.

Two studies have examined breast cancer survival among immigrants in Norway. A small study with data from 2002 to 2009 showed that 63 immigrant women from Pakistan, Sri Lanka and Somalia had more advanced breast cancer and lower survival than Norwegian-born women (Latif et al., 2015). While our study only included women in the age group targeted for mammographic screening, another nationwide, registry-based study with data from 1990 to 2014 included women of all age groups (Thogersen et al., 2017). The study revealed that immigrants from Eastern Europe, the Middle East, Southern Asia and Sub-Saharan Africa had more advanced breast cancer than non-immigrants. Using the same study population, the authors remarked that immigrants from sub-Saharan Africa and immigrants with short duration of residence in Norway had poorer breast cancer survival than non-immigrants (Thøgersen et al., 2018). Further, no statistically significant differences between most immigrant subgroups and non-immigrants were observed. They even found that breast cancer survival was potentially better for non-Western immigrants after including stage at diagnosis as a mediator.

Our findings of more advanced disease but no statistically significant differences in survival among immigrants from non-Western countries versus non-immigrants are in line with the findings from the registry-based study described above. The limited number of breast cancer deaths and shorter follow-up time for immigrants versus non-immigrants, particularly those from non-Western countries, likely represents a bias overestimating survival among immigrants.

The healthy migrant effect may also influence our findings, as immigrants may be healthier than the native population in the country they migrate to (Domnich et al., 2012). Our findings may be further explained by stage distribution within potentially curable stages. We did not find any difference in the proportion of women with incurable stage IV cancers between birth country groups. Among potentially curable stage I–III cancers, we found larger tumors and higher proportions of grade 3 and lymph node positive breast cancers among non-Western immigrants versus non-immigrants. Stage I–III consists of a heterogenous group of breast cancers with a wide range of histopathological factors. Recommendations for treatment of stage I–III breast cancer differ according to histopathological factors, from no treatment other than surgery and radiation therapy for small, lymph node negative luminal-A like tumors, to extensive multimodal treatment for larger tumors with less favorable prognostic markers (Norwegian Directorate of Health 2022). The more advanced breast cancer observed within potentially curable stages of the disease among non-Western immigrants may result in more extensive treatment, as seen with non-Western immigrant groups in Norway less often receiving breast-conserving surgery (which we assume means more often mastectomy) and more often receiving adjuvant chemotherapy than non-immigrants (Thøgersen et al., 2020). More advanced breast cancer in potentially curative stages of the disease among non-Western immigrants rather than a higher proportion of incurable stage IV disease, could partly explain why we did not find lower breast cancer survival among non-Western immigrants despite more advanced disease. However, this does not give a full explanation of our findings, and our study includes a low number of cancer deaths among immigrants.

In our initial interpretation of the results, we theorized that delayed registration of death due to salmon bias could influence our results. Our theory was that women might migrate back to their country of origin after receiving a breast cancer diagnosis, leading to a lack of registration of death in Norway. However, Statistics Norway have investigated this issue and state that people with no known whereabouts are registered as emigrated after two years, meaning that lack of reporting about emigration leads to delayed registration rather than missing registration (Vassenden, 2015). This may overestimate follow-up time and survival among immigrants.

Breast cancer-specific survival among immigrants is a complex and heterogenous issue, with studies showing diverging results. Some studies from other Western countries have shown no difference in or worse breast cancer survival among non-Western immigrants compared to non-immigrants (Norredam et al., 2014; Van Hemelrijck et al., 2020). Potential explanations include lower screening participation and more advanced stage at diagnosis. Other studies, however, have shown better breast cancer survival among groups of non-Western immigrants compared to the host population. Suggested explanations for the better survival include the healthy migrant effect, lifestyle factors, diet and reproductive factors (Anikeeva et al., 2012; Spallek et al., 2012). In order to improve breast cancer survival, strategies focusing on modifiable risk factors for breast cancer should target both immigrants and non-immigrants. Immigrants have been shown to have lower incidence of breast cancer compared to non-immigrants, but their descendants do not, indicating risk adaptation (Beiki et al., 2012; Ziegler et al., 1993), which could potentially reflect a shift in distribution of modifiable risk factors. If so, we can expect similar survival rates for descendants of immigrants as for non-immigrants.

Non-Western immigrants are found to have a lower age at diagnosis (Thogersen et al., 2017). The younger age at diagnosis could potentially mean less frailty and better ability to tolerate treatment. Tumor characteristics may differ according to country of origin or ethnicity (Preat et al., 2014; Plasilova et al., 2016), as also shown in our study. Keeping these findings in mind, we would like future studies to explore whether biological, genetic or epigenetic factors may contribute to no difference in survival despite more advanced disease among non-Western immigrants compared to non-immigrants.

Our finding of no difference in breast cancer-specific survival among immigrants from non-Western countries versus non-immigrants could suggest equity in access for immigrants from non-Western countries despite lower screening attendance and more advanced disease (Thogersen et al., 2017; Bhargava et al., 2017; Thy et al., 2022). One could thus argue that BreastScreen Norway ensures equity in access in keeping with SDG 3 and the Nordic welfare model. However, our results must be interpreted with caution due to the low number of breast cancer deaths, heterogeneity of tumor characteristics and possible overestimation of survival among immigrants. It is further possible that increased screening uptake among immigrants from non-Western countries could lead to earlier detection of breast cancer and perhaps preferential breast cancer-specific survival among immigrants from non-Western countries versus non-immigrants. Finally, differences in survival between immigrants and non-immigrants observed in other studies suggest that variation in survival does exist (Latif et al., 2015; Thøgersen et al., 2018). The low number of cancer deaths does not enable us to explore survival in sub-groups among immigrants from non-Western countries.

Our study uses individual, registry-based data of high quality for the entire target population over a long period of time. The main limitations of this study are related to heterogeneity within the immigrant population, different follow-up times between birth country groups and the low number of cancer cases among immigrants, especially after stratification by detection mode. While the proportion of immigrants in the total population is increasing and breast cancer is the most common type of cancer among women, the absolute number of cancer cases when dividing into subgroups of immigrants is low. We thus divided immigrants into two major groups, those from non-Western countries and those from Western countries, rather than dividing immigrants into continents or smaller regions. There may be major differences in tumor characteristics and survival within these groups. The studies discussed above underscore the importance of considering the influence of differences in composition of immigrant groups, time periods studied and countries/settings. Keeping these limitations in mind, our study is important as it, as far as we know, is the first study exploring survival among individuals in the age group targeted by an organized breast cancer screening program stratified by birth country groups and detection modes.

## 5 Conclusion

We did not find statistically significant differences in breast cancer-specific survival among immigrants from non-Western countries compared to non-immigrants, despite more advanced breast cancer among immigrants from non-Western countries. This could suggest that adequate treatment compensates for more advanced disease, leading to equal outcome in keeping with SDG 3. Low number of breast cancer deaths and possible overestimation of survival among immigrants limit our findings. Service providers should strive to increase attendance among immigrants from non-Western countries in breast cancer screening and explore whether increased attendance could lead to earlier detection of cancer, and thus perhaps better breast cancer-specific survival among immigrants from non-Western countries versus non-immigrants. Policy makers and service providers in other countries can learn from results from our study in order to increase equity in access to screening and treatment of breast cancer.

## Funding

This research did not receive any specific grant from funding agencies in the public, commercial, or not-for-profit sectors.

## Declaration of competing interest

The authors declare the following financial interests/personal relationships which may be considered as potential competing interests:

SB is due to receive personal fees from Gilead outside the submitted work. Apart from this, the authors declare that they have no known competing financial interests or personal relationships that could have appeared to influence the work reported in this paper.

## Appendix Supplementary materials

Image, application 1
